# Supplementary material for: Resolving the full spectrum of human genome variation using Linked-Reads
Source: Genome Res. 2019 Apr;29(4):635–45. doi: 10.1101/gr.234443.118 (PMC6442396; doi:10.1101/gr.234443.118)
Supplement: Supplemental Material [file supp_gr.234443.118_Supplemental_longranger-2.2.zip › longranger-2.2.tar.bz2!/lr2.2-public/lib/stan/cmdstan/stan/doc/api/html/wishart_8hpp_source.html]

Stan: src/stan/prob/distributions/multivariate/continuous/wishart.hpp Source File


|  |  |
| --- | --- |
|  | Stan  2.5.0  probability, sampling & optimization |


- Main Page
- Related Pages
- Namespaces
- Classes
- Files

- File List
- File Members

All Classes Namespaces Files Functions Variables Typedefs Enumerations Enumerator Friends Macros Pages

- src
- stan
- prob
- distributions
- multivariate
- continuous

wishart.hpp

Go to the documentation of this file.

1 #ifndef STAN\_\_PROB\_\_DISTRIBUTIONS\_\_MULTIVARIATE\_\_CONTINUOUS\_\_WISHART\_HPP

2 #define STAN\_\_PROB\_\_DISTRIBUTIONS\_\_MULTIVARIATE\_\_CONTINUOUS\_\_WISHART\_HPP

3

4 #include <boost/concept\_check.hpp>

5

6 #include <stan/agrad/rev/matrix.hpp>

7 #include <stan/math/error\_handling.hpp>

8 #include <stan/math/matrix\_error\_handling.hpp>

9 #include <stan/math/error\_handling/matrix/check\_ldlt\_factor.hpp>

10 #include <stan/math/functions/lmgamma.hpp>

11 #include <stan/math/matrix/crossprod.hpp>

12 #include <stan/math/matrix/columns\_dot\_product.hpp>

13 #include <stan/math/matrix/trace.hpp>

14 #include <stan/math/matrix/log\_determinant\_ldlt.hpp>

15 #include <stan/math/matrix/mdivide\_left\_ldlt.hpp>

16 #include <stan/math/matrix/dot\_product.hpp>

17 #include <stan/math/matrix/mdivide\_left\_tri\_low.hpp>

18 #include <stan/math/matrix/multiply\_lower\_tri\_self\_transpose.hpp>

19 #include <stan/math/matrix/meta/index\_type.hpp>

20 #include <stan/prob/constants.hpp>

21 #include <stan/prob/traits.hpp>

22 #include <stan/prob/distributions/univariate/continuous/normal.hpp>

23 #include <stan/prob/distributions/univariate/continuous/chi\_square.hpp>

24

25 namespace stan {

26

27  namespace prob {

28

29  // Wishart(Sigma|n,Omega) [Sigma, Omega symmetric, non-neg, definite;

30  // Sigma.dims() = Omega.dims();

31  // n > Sigma.rows() - 1]

59  template <bool propto,

60  typename T\_y, typename T\_dof, typename T\_scale>

61  typename boost::math::tools::promote\_args<T\_y,T\_dof,T\_scale>::type

62  wishart\_log(const Eigen::Matrix<T\_y,Eigen::Dynamic,Eigen::Dynamic>& W,

63  const T\_dof& nu,

64  const Eigen::Matrix<T\_scale,Eigen::Dynamic,Eigen::Dynamic>& S) {

65  static const char\* function = "stan::prob::wishart\_log(%1%)";

66

67  using boost::math::tools::promote\_args;

68  using Eigen::Dynamic;

69  using Eigen::Lower;

70  using Eigen::Matrix;

71  using stan::math::check\_greater;

72  using stan::math::check\_ldlt\_factor;

73  using stan::math::check\_size\_match;

74  using stan::math::index\_type;

75  using stan::math::LDLT\_factor;

76  using stan::math::log\_determinant\_ldlt;

77  using stan::math::mdivide\_left\_ldlt;

78

79

80  typename index\_type<Matrix<T\_scale,Dynamic,Dynamic> >::type k

81  = W.rows();

82  typename promote\_args<T\_y,T\_dof,T\_scale>::type lp(0.0);

83  check\_greater(function, nu, k-1,

84  "Degrees of freedom parameter", &lp);

85  check\_size\_match(function,

86  W.rows(), "Rows of random variable",

87  W.cols(), "columns of random variable",

88  &lp);

89  check\_size\_match(function,

90  S.rows(), "Rows of scale parameter",

91  S.cols(), "columns of scale parameter",

92  &lp);

93  check\_size\_match(function,

94  W.rows(), "Rows of random variable",

95  S.rows(), "columns of scale parameter",

96  &lp);

97  // FIXME: domain checks

98

99  LDLT\_factor<T\_y,Eigen::Dynamic,Eigen::Dynamic> ldlt\_W(W);

100  if (!check\_ldlt\_factor(function,ldlt\_W,

101  "LDLT\_Factor of random variable",&lp))

102  return lp;

103

104  LDLT\_factor<T\_scale,Eigen::Dynamic,Eigen::Dynamic> ldlt\_S(S);

105  if (!check\_ldlt\_factor(function,ldlt\_S,

106  "LDLT\_Factor of scale parameter",&lp))

107  return lp;

108

109  using stan::math::trace;

110  using stan::math::lmgamma;

111  if (include\_summand<propto,T\_dof>::value)

112  lp += nu \* k \* NEG\_LOG\_TWO\_OVER\_TWO;

113

114  if (include\_summand<propto,T\_dof>::value)

115  lp -= lmgamma(k, 0.5 \* nu);

116

117  if (include\_summand<propto,T\_dof,T\_scale>::value)

118  lp -= 0.5 \* nu \* log\_determinant\_ldlt(ldlt\_S);

119

120  if (include\_summand<propto,T\_scale,T\_y>::value) {

121  Matrix<typename promote\_args<T\_y,T\_scale>::type,Dynamic,Dynamic>

122  Sinv\_W(mdivide\_left\_ldlt(ldlt\_S,

123  static\_cast<Matrix<T\_y,Dynamic,Dynamic> >(W.template selfadjointView<Lower>())));

124  lp -= 0.5 \* trace(Sinv\_W);

125  }

126

127  if (include\_summand<propto,T\_y,T\_dof>::value && nu != (k + 1))

128  lp += 0.5 \* (nu - k - 1.0) \* log\_determinant\_ldlt(ldlt\_W);

129  return lp;

130  }

131

132  template <typename T\_y, typename T\_dof, typename T\_scale>

133  inline

134  typename boost::math::tools::promote\_args<T\_y,T\_dof,T\_scale>::type

135  wishart\_log(const Eigen::Matrix<T\_y,Eigen::Dynamic,Eigen::Dynamic>& W,

136  const T\_dof& nu,

137  const Eigen::Matrix<T\_scale,Eigen::Dynamic,Eigen::Dynamic>& S) {

138  return wishart\_log<false>(W,nu,S);

139  }

140

141  template <class RNG>

142  inline Eigen::Matrix<double, Eigen::Dynamic, Eigen::Dynamic>

143  wishart\_rng(const double nu,

144  const Eigen::Matrix<double,Eigen::Dynamic,Eigen::Dynamic>& S,

145  RNG& rng) {

146

147  using Eigen::MatrixXd;

148  using stan::math::index\_type;

149  using stan::math::check\_size\_match;

150  using stan::math::check\_positive;

151

152  static const char\* function = "stan::prob::wishart\_rng(%1%)";

153

154  typename index\_type<MatrixXd>::type k = S.rows();

155

156  check\_positive(function,nu,"degrees of freedom",(double\*)0);

157  check\_size\_match(function,

158  S.rows(), "Rows of scale parameter",

159  S.cols(), "columns of scale parameter",

160  (double\*)0);

161

162  MatrixXd B = MatrixXd::Zero(k, k);

163

164  for (int j = 0; j < k; ++j) {

165  for (int i = 0; i < j; ++i)

166  B(i, j) = normal\_rng(0, 1, rng);

167  B(j,j) = std::sqrt(chi\_square\_rng(nu - j, rng));

168  }

169

170  return stan::math::crossprod(B \* S.llt().matrixU());

171  }

172

173

174  }

175

176 }

177 #endif

matrix.hpp

mdivide\_left\_tri\_low.hpp

chi\_square.hpp

mdivide\_left\_ldlt.hpp

stan::math::check\_greater

bool check\_greater(const char \*function, const T\_y &y, const T\_low &low, const char \*name, T\_result \*result)

**Definition:** check\_greater.hpp:58

traits.hpp

stan::prob::wishart\_log

boost::math::tools::promote\_args< T\_y, T\_dof, T\_scale >::type wishart\_log(const Eigen::Matrix< T\_y, Eigen::Dynamic, Eigen::Dynamic > &W, const T\_dof &nu, const Eigen::Matrix< T\_scale, Eigen::Dynamic, Eigen::Dynamic > &S)

The log of the Wishart density for the given W, degrees of freedom, and scale matrix.

**Definition:** wishart.hpp:62

multiply\_lower\_tri\_self\_transpose.hpp

lmgamma.hpp

columns\_dot\_product.hpp

stan::math::check\_size\_match

bool check\_size\_match(const char \*function, T\_size1 i, const char \*name\_i, T\_size2 j, const char \*name\_j, T\_result \*result)

**Definition:** check\_size\_match.hpp:13

error\_handling.hpp

crossprod.hpp

stan::math::mdivide\_left\_ldlt

Eigen::Matrix< typename boost::math::tools::promote\_args< T1, T2 >::type, R1, C2 > mdivide\_left\_ldlt(const stan::math::LDLT\_factor< T1, R1, C1 > &A, const Eigen::Matrix< T2, R2, C2 > &b)

Returns the solution of the system Ax=b given an LDLT\_factor of A.

**Definition:** mdivide\_left\_ldlt.hpp:24

matrix\_error\_handling.hpp

constants.hpp

trace.hpp

stan::agrad::mdivide\_left\_ldlt

Eigen::Matrix< fvar< T2 >, R1, C2 > mdivide\_left\_ldlt(const stan::math::LDLT\_factor< double, R1, C1 > &A, const Eigen::Matrix< fvar< T2 >, R2, C2 > &b)

Returns the solution of the system Ax=b given an LDLT\_factor of A.

**Definition:** mdivide\_left\_ldlt.hpp:25

stan::math::check\_ldlt\_factor

bool check\_ldlt\_factor(const char \*function, stan::math::LDLT\_factor< T, R, C > &A, const char \*name, T\_result \*result)

Return true if the underlying matrix is positive definite.

**Definition:** check\_ldlt\_factor.hpp:24

check\_ldlt\_factor.hpp

stan::math::index\_type

Primary template class for the metaprogram to compute the index type of a container.

**Definition:** index\_type.hpp:21

normal.hpp

stan::prob::include\_summand

Template metaprogram to calculate whether a summand needs to be included in a proportional (log) prob...

**Definition:** traits.hpp:35

stan::math::lmgamma

boost::math::tools::promote\_args< T >::type lmgamma(const int k, T x)

Return the natural logarithm of the multivariate gamma function with the speciifed dimensions and arg...

**Definition:** lmgamma.hpp:57

stan::math::LDLT\_factor

**Definition:** LDLT\_factor.hpp:16

stan::agrad::sqrt

fvar< T > sqrt(const fvar< T > &x)

**Definition:** sqrt.hpp:15

log\_determinant\_ldlt.hpp

index\_type.hpp

stan::prob::normal\_rng

double normal\_rng(const double mu, const double sigma, RNG &rng)

**Definition:** normal.hpp:377

stan::math::check\_positive

bool check\_positive(const char \*function, const T\_y &y, const char \*name, T\_result \*result)

**Definition:** check\_positive.hpp:54

dot\_product.hpp

stan::prob::wishart\_rng

Eigen::Matrix< double, Eigen::Dynamic, Eigen::Dynamic > wishart\_rng(const double nu, const Eigen::Matrix< double, Eigen::Dynamic, Eigen::Dynamic > &S, RNG &rng)

**Definition:** wishart.hpp:143

stan::agrad::log\_determinant\_ldlt

var log\_determinant\_ldlt(stan::math::LDLT\_factor< var, R, C > &A)

**Definition:** log\_determinant\_ldlt.hpp:48

stan::math::trace

T trace(const Eigen::Matrix< T, Eigen::Dynamic, Eigen::Dynamic > &m)

Returns the trace of the specified matrix.

**Definition:** trace.hpp:20

stan::math::log\_determinant\_ldlt

T log\_determinant\_ldlt(stan::math::LDLT\_factor< T, R, C > &A)

**Definition:** log\_determinant\_ldlt.hpp:12

stan::agrad::lmgamma

fvar< typename stan::return\_type< T, int >::type > lmgamma(int x1, const fvar< T > &x2)

**Definition:** lmgamma.hpp:16

stan::math::crossprod

matrix\_d crossprod(const matrix\_d &M)

Returns the result of pre-multiplying a matrix by its own transpose.

**Definition:** crossprod.hpp:17

stan::prob::chi\_square\_rng

double chi\_square\_rng(const double nu, RNG &rng)

**Definition:** chi\_square.hpp:442


---

[ Stan Home Page ]

*© 2011–2014,
Stan Development Team.*
